# Exploring the overdoping effects in Transient Liquid Assisted Grown YBa$_2$Cu$_3$O$_{7-\delta }$ superconducting films

**DOI:** 10.1038/s41598-026-41613-0

**Published:** 2026-04-01

**Authors:** Aiswarya Kethamkuzhi, Lavinia Saltarelli, Kapil Gupta, Carla Torres, Diana Garcia, Joffre Gutierrez, Xavier Obradors, Teresa Puig

**Affiliations:** 1 Institute of Materials Science of Barcelona (ICMAB-CSIC), Bellaterra, Catalonia, Spain; 2 Catalan Institute of Nanoscience and Nanotechnology (ICN2), Bellaterra, Catalonia, Spain

**Keywords:** Materials science, Physics

## Abstract

The enhancement of the current-carrying capabilities of cuprate materials depends highly on the knowledge of how to tweak their doping state. The recent efforts on overdoping YBa$_2$Cu$_3$O$_{7-\delta }$ (YBCO) to push the limit of critical current density towards the depairing critical current density are also based on the study of their doping state. We present the overdoping study of Transient Liquid Assisted Grown YBCO films through various oxygenation strategies, including oxygen-ozone mixtures and Ag surface-decoration layers. We systematically analyze how the key kinetic parameters, such as temperature, dwell time, ozone concentration, and surface modification, influence the oxygen content and superconducting properties of the films. Our results provide insights into achieving the overdoped regime in TLAG YBCO and offer a comparison with films grown via the Pulsed Laser Deposition (PLD) method. This study of overdoping on the TLAG YBCO films, which compares three distinct oxygenation methods and conducts an unprecedented analysis of the ozone-assisted process, promises a substantial enhancement in REBa$_2$Cu$_3$O$_{7-\delta }$ (REBCO, RE = rare earth elements) fabrication.

## Introduction

High-temperature superconductors (HTS) are among the most remarkable achievements since the discovery of superconductivity and hold significant potential for transforming large-scale energy applications. Over the past thirty years, considerable efforts have been made to enhance the current-carrying capacity of coated conductors (CC), which has greatly expanded their applicability^1,2^. The fabrication of highly textured CC made it feasible to successfully use REBCO in viable applications^3,4^. However, their potential to transform the energy sector is constrained by the high cost/performance ratio associated with CC fabrication. Increasing the growth rate is one of the primary strategies to lower the cost/performance ratio^5^. High growth rates exceeding 1000 nm/s and a critical current density in the regime of 2-3 MA/cm$^2$ at 77 K are successfully attained by combining the innovative Transient Liquid Assisted Growth (TLAG) method with the conventional Chemical Solution Deposition (CSD) strategy^6–10^. However, the critical current density ($J_c$) remains below its theoretical depairing limit since dissipation from vortex motion starts prior to Cooper pair splitting^3,11^.

In recent years, considerable research has focused on improving the pinning strength of vortices to enhance the performance of CC under magnetic fields. One of the widely used approaches to efficiently pin vortices is the addition of nanoscale defects that serve as artificial pinning centers (APCs)^5,12–15^. Another approach is to explore oxygen doping beyond the optimal doping state (i.e., maximum $T_c$) to reach the overdoped regime, where $T_c$ is slightly reduced but the condensation energy increases, thus boosting $J_c$^16–21^. This method seeks to enhance charge carrier density and push REBCO-CC to the overdoped state, where $J_c$ is maximized^5,14^. Current carrying capacities may even be pushed closer to the depairing current density ($J_d$) by combining these two techniques, according to recent research on overdoped nanocomposites^14,18,21^.

The electronic properties of cuprate superconductors are strongly influenced by the hole doping level, $\textit{p}$, which is determined by the oxygen content in the structure^18^. By overdoping, we increase $\textit{p}$ beyond the optimal doping value $\textit{p}_{opt}=0.16$, to the quantum critical point $\textit{p}^*=0.19$ where the pseudogap energy spectrum vanishes abruptly and the Fermi surface suffers a reconstruction^22^. As the superconducting properties of REBCO are strongly determined by the oxygen doping state, adding oxygen to the lattice is an essential stage in the production process. However, introducing oxygen into the YBCO lattice and controlling the doping level is complex and not yet fully understood^23–26^. The microstructure of YBCO has a significant impact on the ease of oxygen incorporation, with surface activation being a critical, rate-limiting step in this process^14,27^^24^ and grain boundaries of coated conductors being a route to accelerate oxygenation^28^. Reaching the overdoped state of single crystals and CC is supported by low-temperature oxygenation processes^29^; but the challenge of slow diffusion at such low temperatures needs to be addressed^23,24^. In order to improve oxygen incorporation, we have investigated the overdoped state in TLAG YBCO films utilizing several oxygenation techniques.

Particularly, it is known that annealing HTS films with ozone is a successful strategy to increase the oxygen content in these materials, even at low temperatures, due to its high oxidation power, thus allowing to stabilize novel HTS crystal structures^30^ to reach higher values of overdoping^31–33^ or improve the quality of HTS nanostructures^34^. We have, therefore, explored this strategy to reach the overdoped state in our YBCO films. A second strategy has been the use of a silver surface-decoration layer, a technique previously shown to facilitate surface diffusion^18^.

Being all these approaches kinetically controlled processes, temperature, dwell time, and ozone concentration (in the case of the oxygen-ozone mixture process) all have a significant impact on the final oxygen content of the material. This study offers a thorough analysis of the major oxygenation parameters that affect the characteristics of TLAG YBCO films. We carefully examine how these parameters affect the achievement of the intended overdoped state. Furthermore, this study examines the properties of the overdoped state in TLAG YBCO pristine and nanocomposite films in particular, emphasizing how their critical current density and other physical properties vary from the overdoped films prepared from the PLD method^18^. Overall, the main aim of this study is to demonstrate the capability of TLAG YBCO films in achieving the overdoped state.

## Results

The YBCO films used in the oxygenation study are prepared via the Transient Liquid Assisted Growth combined with the chemical solution deposition method. All the pristine films are composed of two pyrolyzed layers deposited on SrTiO$_3$ (STO) substrate with the final thickness around 400 nm after growth. The nanocomposite films are composed of a thin pristine seed layer followed by two nanocomposite layers and a thin pristine cap layer with a total sample thickness around 625 nm.

The three main oxygenation methods under exploration are conventional oxygen annealing (TLAG oxygen), ozone-assisted annealing (TLAG ozone), and oxygenation through silver (Ag) island surface decorative layer on top of the YBCO film (TLAG Ag). We have annealed each YBCO film at one specific temperature for 60 min only and slowly cooled them down to room temperature at a rate of $10\,^\circ \textrm{C}$/min. As each annealing treatment was performed on a separate film, only reproducible and epitaxial YBCO films, as confirmed by X-ray diffraction (XRD) measurements, were used in the experiments. The degree of sample-to-sample variability is reported in the supplementary information Figure S1.

In the case of TLAG Ag, the catalytic effect of silver during oxygenation of YBCO has been previously proposed^35,36^ and is widely used by CC manufacturers. The rate-limiting step in the oxygenation process is the surface reaction, wherein the oxygen atoms are incorporated. Silver participates in the dissociation of the oxygen molecule into individual oxygen atoms, which can subsequently be absorbed by the surface^21,23^. This catalytic effect of silver enhances the oxygen kinetics at low temperatures and reduces the time to achieve the desired doping state. We adapted the same procedure used by A. Stangl et al.^27^, where 100 nm Ag squares are deposited on top of YBCO films before oxygen annealing at temperatures between 400 $^\circ \textrm{C}$ and 500 $^\circ \textrm{C}$ for 60 min.

Unlike the widely explored Ag surface decoration method, there are very few examples^37,38^ of ozone being used for YBCO oxygenation,even though it has been shown in other HTS materials that it can play a beneficial role in reaching high oxidation states^31–33^. The main advantage of using an ozone-oxygen mixture is its highly oxidizing nature, thereby keeping the partial pressure of oxygen atoms at the surface of YBCO high, which is readily absorbed by the surface. However, some preliminary studies of thin films or bulk ceramics already indicated that care had to be taken to avoid the chemical or structural degradation of the YBCO and Ag protection layers of CC when excessively high oxidizing conditions are used which promote the formation of stacking faults of complex structure^39–41^.

Therefore, it is necessary to fine-tune the conditions to use ozone for getting the desired oxygenation results without deteriorating the films. The tuning was carried out by adjusting the temperature, dwell time, and ozone concentration. The optimal conditions are primarily determined by evaluating the percolative critical current density ($J_c^{\text {GB}}$), with further validation provided by calculating the grain critical current density ($J_c^{\text {Grain}}$) determined from minor loop measurements, following the same analysis by A. Palau et al.^42^ which is also explained in the supplementary information Figure S2. This method enables us to distinguish zones of maximum critical current density ($J_c^{\text {Grain}}$) developing local loops caused by non-uniform oxygenation from the overall percolative critical current density of the film ($J_c^{\text {GB}}$) calculated from magnetization measurements using Bean critical state model explained in the supplementary information Figure S3. For that purpose, all TLAG films in this study were grown under the same process conditions and thoroughly analyzed by XRD and scanning electron microscopy (SEM) prior to the oxygenation treatment. This ensured that the percolative $J_c$ was not affected by standard granularity effects caused by grain misalignments, extensive porosity, or bad connectivity of grains^43,44^.

### Optimization of ozone-assisted oxygenation

**Fig. 1:**
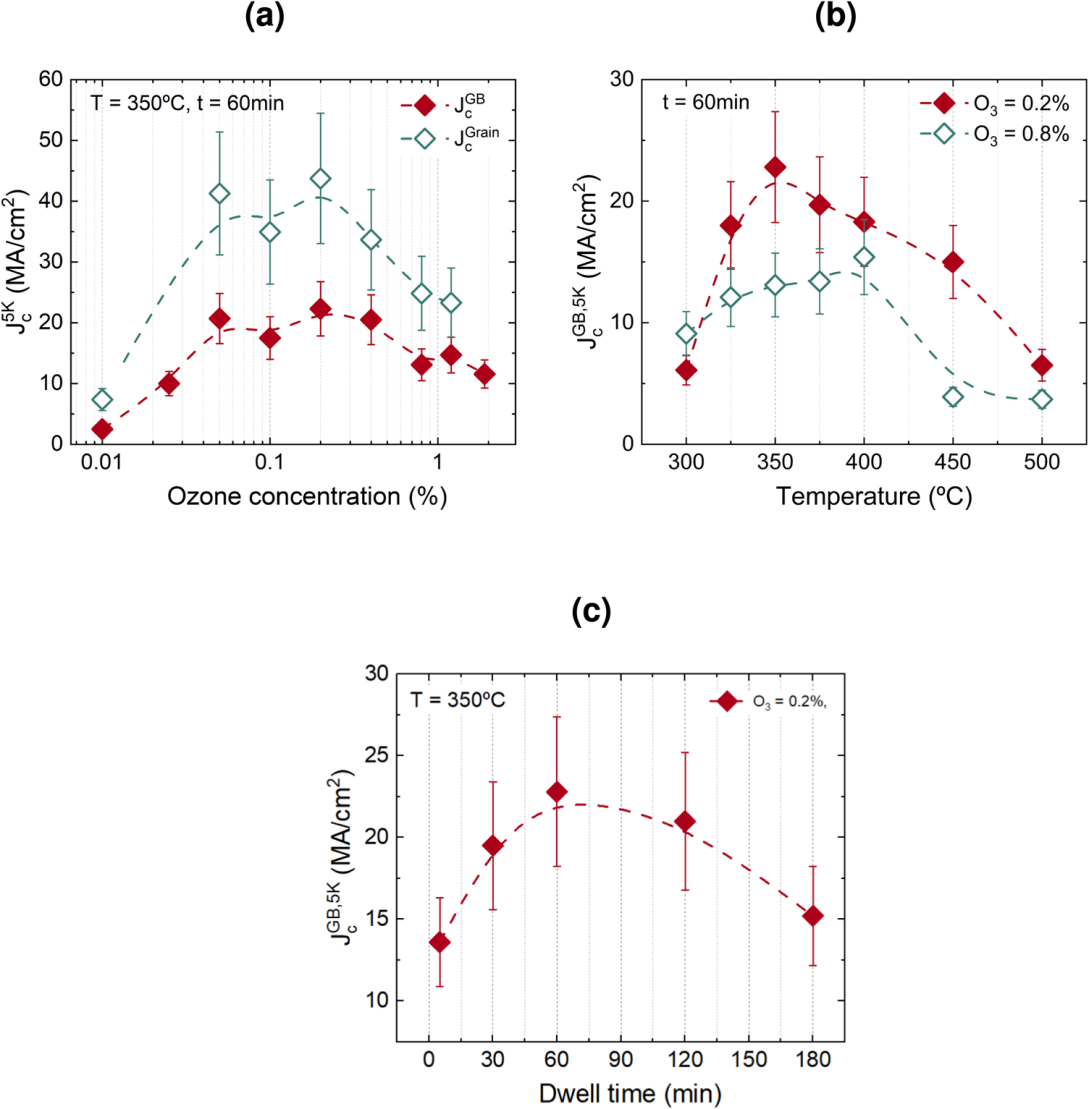
Optimization of ozone-assisted oxygenation: (**a**) percolation critical current density and grain critical current density as a function of ozone concentration, (**b**) $J_c^{\text {GB}}$ as a function of annealing temperature for two different concentrations of ozone, (**c**) $J_c^{\text {GB}}$ as a function of dwell time.

In our experimental setup for ozone-assisted oxygenation, the gas mixture flows through the system with a total flow rate of 0.2 L/min at 1 bar pressure. The amount of ozone in the mixture is expressed in terms of volume percentage, and the range of achievable ozone concentrations within our system varied from as low as 0.01 % to as high as 1.9 %. The gas mixture was allowed to flow through the furnace with the sample placed inside for 10-15 minutes before the heat treatment began. To identify the optimal concentration for enhancing the critical current density $J_c$, we systematically varied the ozone concentration as shown in Figure 1a when the annealing temperature is $350\,^\circ \textrm{C}$. At very low ozone concentrations, the critical current density remains low, which indicates that the oxidation kinetics at such a low ozone concentration is still too slow to effectively complete the oxygen incorporation reaction.

As the concentration increases, we achieve good-quality YBCO films with high critical current density. We observed that there is a region of ozone concentration from 0.05 %-0.2 % where the percolation and grain critical current densities are maximum. In this range of concentration, ozone is sufficient to catalyze the reaction without causing adverse effects on the crystalline quality. Beyond this optimal range of concentration, there is a decrease in $J_c$ values, indicating enhanced degradation of the films. To disentangle the origin of this degradation, a microstructural analysis of the films was required. We first observed a gradual change in the surface morphology of the films through SEM study shown in the supporting information Figure S4. However, an analysis of the YBCO (00*l*) peaks in the XRD patterns shown in Figure S5 showed a reduced intensity which can only be understood as arising from some bulk degradation of the structure. The surface deteriorates more as the concentration rises due to the strong oxidising nature of ozone, likely leading to the formation of a surface dead layer that may partially or fully lose its superconducting properties. Overall, based on these observations, we conclude that the optimal range for working with ozone for maximizing the critical current density and preserving the structural quality is between 0.05 %- 0.2 %.

The second important parameter in the optimization process is the annealing temperature. We performed annealing at different temperatures for 60 min, followed by slow cooling to room temperature with a rate of 10 $^\circ \textrm{C}$/min, and evaluated the superconducting properties to determine the right temperature for using ozone. The resulting percolation critical current density for the films treated with two different ozone concentrations (0.2 % and 0.8 %) is depicted in Figure 1b . It is observed that below $350\,^\circ \textrm{C}$, $J_c$ decreases probably due to slow kinetics at such low temperatures^24^. Whereas, at elevated temperatures, the highly reactive nature of ozone leads to degradation of the film’s quality. The overall reduction of $J_c$ when using higher concentrations of ozone throughout the temperature range is also shown in Figure 1b (open symbols).

The third parameter that we explored is the dwell time of oxygenation, which is shown in Figure 1c . The highest $J_c$ is observed when the dwell time is around 60 min for ozone-treated films at $350\,^\circ \textrm{C}$ with 0.2 % ozone. Shorter dwell times result in incomplete oxygen incorporation, while longer dwell times decrease $J_c$ probably due to prolonged exposure to the highly reactive ozone environment. From the fine-tuning of ozone-assisted oxygenation, we found that ozone concentration in the range of 0.05 %-0.2 % and annealing at $350\,^\circ \textrm{C}$ for 60 min generate good-quality YBCO films without destroying the crystalline quality.

### Comparison of oxygenation methods

**Fig. 2:**
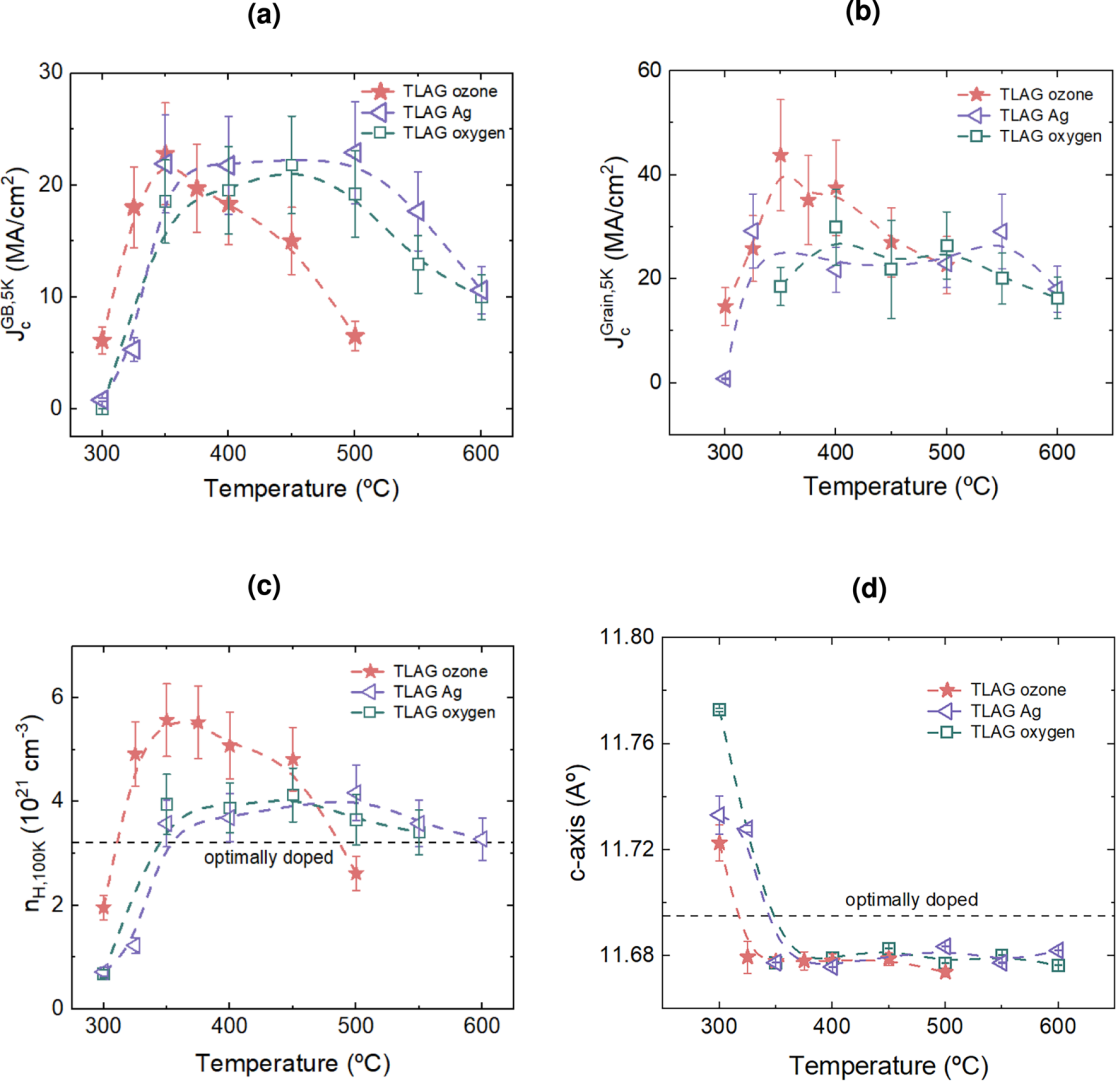
Comparison of oxygenation methods: Evolution of (**a**) percolation critical current density, $J_c^{\text {GB}}$ measured by SQUID magnetometer, and (**b**) grain critical current density, $J_c^{\text {Grain}}$ calculated from minor loops followed by the method in ^42^ at 5 K as a function of the annealing temperature. Samples oxygenated under different oxygenation methods are depicted with different symbols. Their corresponding (**c**) Hall carrier density $n_H$ at 100 K and (d) *c*-axis parameter as a function of annealing temperature are also presented.

In Figure 2, we compare the results of TLAG YBCO films annealed using the three above-mentioned oxygenation methods at temperatures ranging from $350\,^\circ \textrm{C}$ to $550\,^\circ \textrm{C}$ for 60 min followed by a controlled cooling process to room temperature at a rate of 10 $^\circ \textrm{C}/$min. In the ozone-assisted process, the concentration of ozone used was 0.2%. The percolation and grain critical current density as a function of annealing temperature is shown in Figures 2a and 2b . The oxygen atoms diffuse into the YBCO lattice through regions of high diffusivity, and this creates a network of electromagnetic grain and grain boundaries. The percolation critical current density associated with these grain boundaries will limit the overall superconducting properties when the oxygenation is not homogeneous. However, we are interested in the actual effect of overdoping reflected in the grains. Therefore, distinguishing between the percolation critical current density and the grain critical current density is essential for understanding how oxygenation can promote overdoping^27,45^.

In all three types of YBCO films, the critical current densities display a dome-shaped behavior with the annealing temperature. In the case of TLAG ozone, the maximum of the percolation and grain critical current density is observed at $350\,^\circ \textrm{C}$. For TLAG oxygen and Ag films, the high percolation critical current density region is broader and is between $350\,^\circ \textrm{C}$ - $500\,^\circ \textrm{C}$, but the maximum grain critical current density occurs beyond $350\,^\circ \textrm{C}$. The difference in oxygenation capability of the three methods is reflected in the grain properties. Especially ozone is able to achieve higher critical current density of grains compared to the other two methods.

The decrease of $J_c$ beyond $350\,^\circ \textrm{C}$ for ozone films is might be due to the detrimental effect caused by the high reactivity of ozone at high temperatures. The trend in $J_c$ is further corroborated by the variations in the *c*-axis lattice parameter and the Hall carrier density ($n_H$), as shown in Figures 2c and 2d . Carrier density $n_H$ is obtained from Hall measurements, which quantify the total number of charge carriers per unit volume associated with the charges present in the CuO chains and CuO$_2$ planes, and the *c*-axis parameter is extracted from the XRD pattern by Nelson-Riley analysis^46^. Both of these quantities can be retrieved rather simply and are directly impacted by changes in the oxygen content of the YBCO lattice. At low annealing temperatures, the *c*-axis parameter remains large, and the carrier density is rather low, indicating that the oxygenation process was not fully completed. As a result, the films remain in the underdoped state, below the optimal doping level defined by the known $n_H$ value for the optimally doped state^18^, $3.2\times 10^{21} cm^{-3}$.

**Fig. 3:**
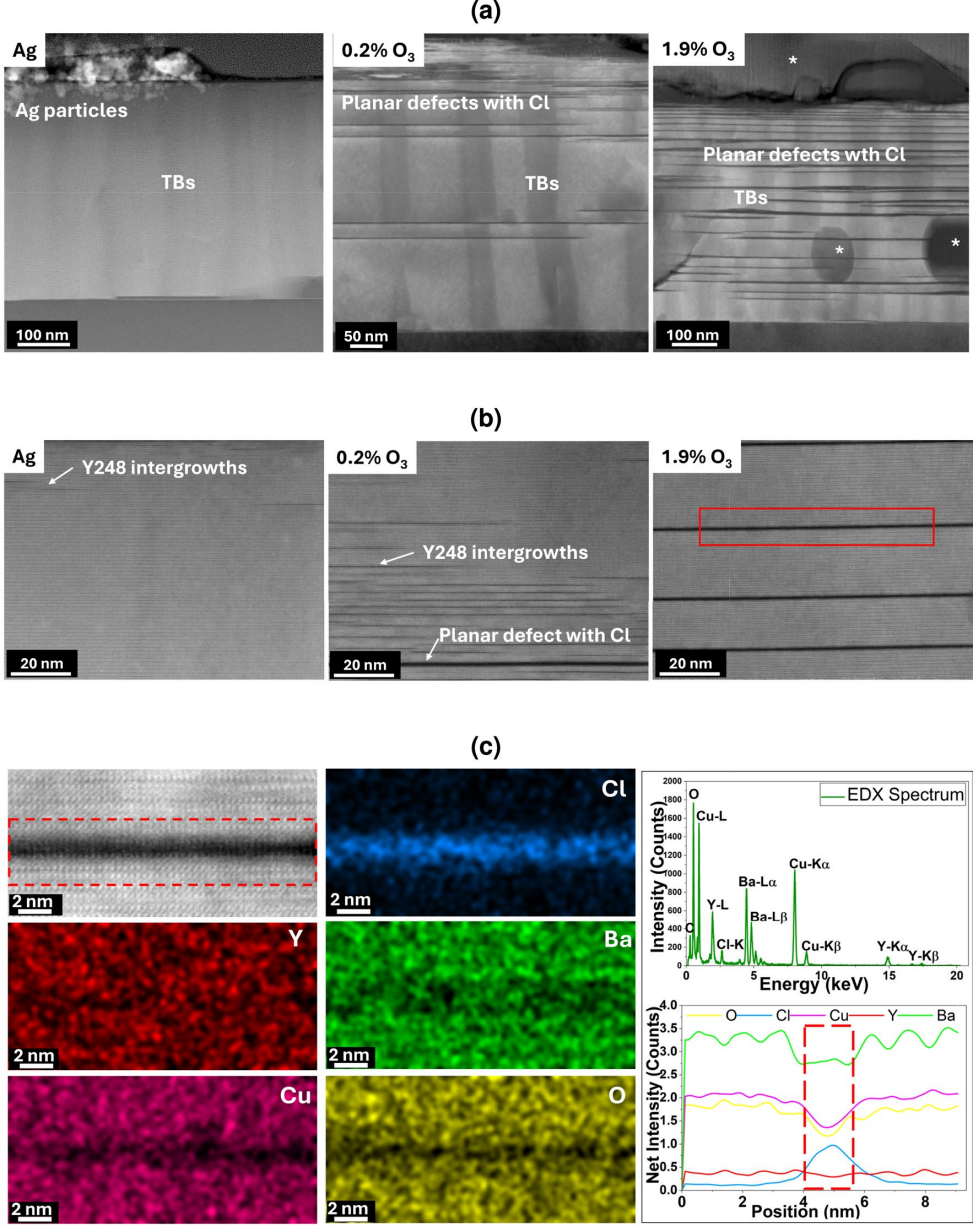
Microstructure comparison between TLAG Ag films and TLAG ozone films: (**a**) Low-magnification STEM-High Angle Annular Dark Field (HAADF) images of cross-sectional lamellae from a YBCO film oxygenated with an Ag layer and two YBCO films oxygenated with 0.2% and 1.9% ozone. Secondary phases are indicated by asterisks. The most commonly occurring secondary phases are CuO (on the top), some Ba-Cu-O phase (eg: BaCu$_3$O$_4$), Y211 phase (Y$_2$BaCuO$_5$), Y$_2$O$_3$, etc. (**b**) Atomic-resolution STEM-HAADF images reveal two types of planar defects: Y$_2$Ba$_4$Cu$_8$O$_x$ (Y248) intergrowths and defects associated with a chlorine-rich phase. (**c**) The EDX analysis on the planar defect; the presence of chlorine along with barium is observed, suggesting some Ba-Cl phase formation.

As the temperature is increased to $350\,^\circ \textrm{C}$, the *c*-axis parameter reduces significantly to less than 11.695Å, which is considered to be the optimal *c*-axis parameter for YBCO. A complementary behavior is observed in $n_H$ values, increasing to more than two times the value in the underdoped state. These two parameters together confirm oxygen incorporation into the YBCO lattice. As the temperature is increased beyond $450\,^\circ \textrm{C}$, the $n_H$ values of TLAG ozone films decrease. However, TLAG oxygen and Ag films remain in the optimally doped range. We attribute this decrease in TLAG ozone films to the deterioration of the YBCO surface and/or bulk structure associated to the highly reactive nature of ozone, which introduces uncertainty in the determination of $n_H$. The lattice parameter remains unaffected in all these films when the annealing temperature is increased, which confirms that the decrease observed in $n_H$ with ozone at high temperatures might be associated to changes in the electronic structure or the transport properties associated to the generated defects.

Insights into the origin of the observed degradation of the surface and bulk crystalline structure, microstructure, and superconducting properties of ozone-annealed YBCO films is obtained by comparing the microstructures of YBCO films oxygenated with Ag and ozone as shown in Figure 3a and 3b .The TLAG Ag film contains mainly coherent twin boundaries (TBs) and a few Y$_2$Ba$_4$Cu$_8$O$_x$ (Y248) intergrowths. On the other hand, TLAG ozone reveals two types of planar defects; Y248 intergrowths and thick planar defects associated with a chlorine-rich phase, as confirmed by Energy Dispersive X-ray (EDX) spectroscopy analysis shown in Figure 3c . The density of planar defects related to the chlorine-rich phase increases with higher ozone concentration and as we mentioned before, this decreases the intensity of (00*l*) Bragg peaks (supplementary information Figure S5). This is very likely related to the stacking disorder generated by the planar defects which then generate a strong X-ray diffuse scattering, similarly to what it was previously observed in CSD-Trifluoroacetate grown YBCO films of small thickness where a large concentration of Y124 stacking fault planar defects were generated^47^.The presence of twin boundaries is also observed in TLAG ozone films. However, their coherence is broken by the thick planar defects. The chlorine contamination likely was originated from the tubes used to transport the oxygen-ozone mixture. At this stage, therefore, we attribute the decrease of $J_c$ beyond $350\,^\circ \textrm{C}$ for ozone films mainly to the formation of these planar defects caused by the ozone treatments.

We have demonstrated clear pathways to enhance the oxygen doping state in terms of $n_H$ higher than $3.2\times 10^{21} cm^{-3}$, which is considered as the optimal charge carrier density corresponding to $\textit{p}_{opt} = 0.16$. This enhancement together with the changes in *c*-axis lattice parameter, evidences higher doping states in TLAG YBCO films. A notable increase in charge carrier density is observed in TLAG ozone films compared to those produced by the other two processes, particularly at relatively low temperatures such as $350\,^\circ \textrm{C}$. This highlights ozone’s role in enhancing oxygenation kinetics; however, some degradation may have occurred in ozone-treated films, especially at high temperatures or large ozone concentrations, which could negatively affect the critical current density.

### Doping state analysis of TLAG films

We utilize several parameters, such as $n_H$, $T_c$, *c*-axis, and normal state resistivity, to assess the oxygenation process and determine the doping state of the films. The distinction of the doping state in the overdoped regime is complex and cannot be determined from using only one of the parameters mentioned above. Therefore, with the combined analysis of these parameters, we calculated $\textit{p}$ to distinguish various TLAG YBCO films. For underdoped films, $\textit{p}$ is calculated from $T_c$ following the conventional cuprate relationship, ${1-T_c/T_{c,max}=82.6(p-0.16)^2}$ with $T_{c,max}$ = 92 K. Whereas for optimally doped and overdoped films, we extract $\textit{p}$ with the help of the *c*-axis using an empirical formula as explained in the methods. The $n_H$ value was the criterion to decide whether the YBCO film is underdoped, optimally doped, or overdoped, by the same method followed by Miura et al.^21^.

**Fig. 4:**
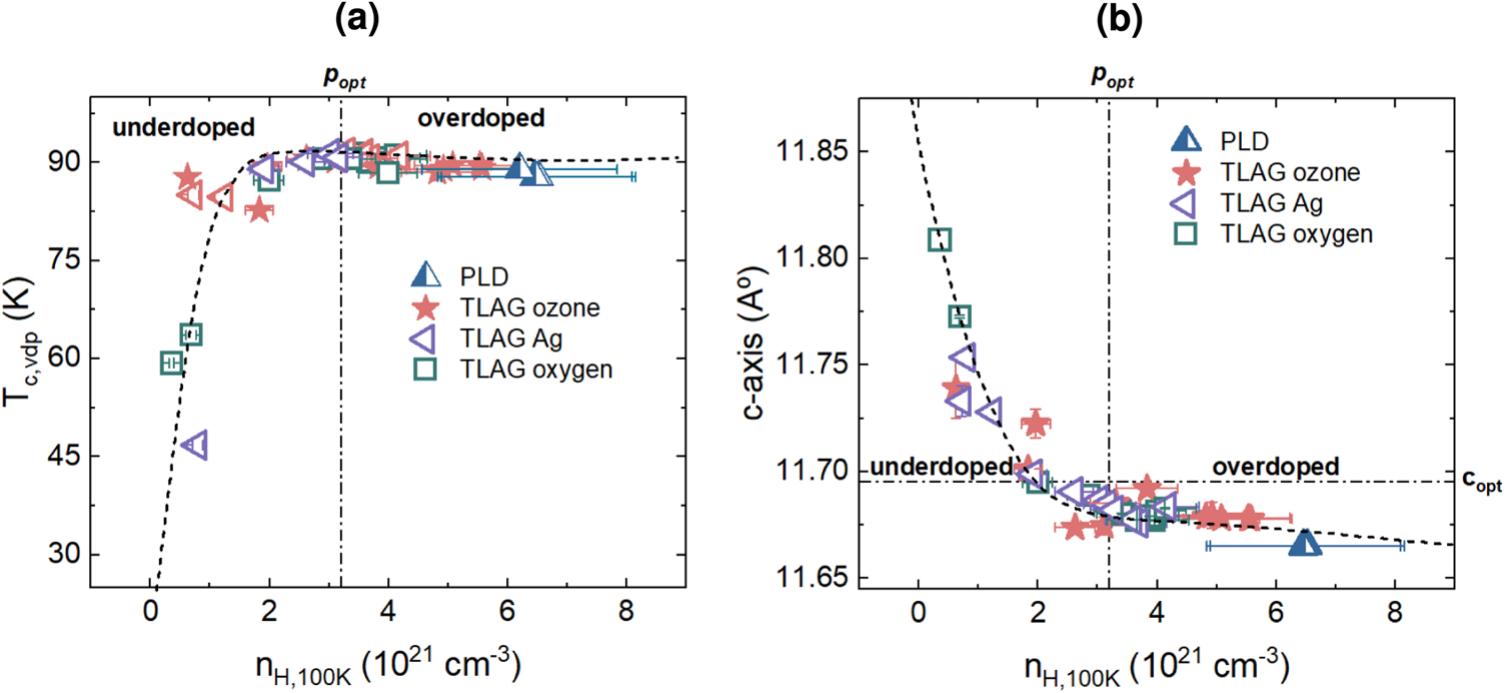
Doping state comparison between TLAG and PLD YBCO films: Dependence of (**a**) critical temperature $T_c$ and (**b**) *c*-axis parameter on Hall carrier density $n_H$ of films grown via TLAG and PLD methods. The data of PLD films were reproduced from A. Stangl et al.^18^. Different methods of oxygenation (oxygen, Ag, ozone) are depicted using different symbols.For the TLAG ozone films, concentration of ozone used is between 0.05-0.2%.The optimal doping point corresponding to $\textit{p}_{opt} = 0.16$ is depicted as a vertical line in both figures; the horizontal line in (**b**) corresponds to the optimal *c*-axis value of 11.695Å. The dashed line following the data points is a guide to the eye.

The mutual relationship between $T_c$, $n_H$, and *c*-axis is shown in Figure 4. The doping state of TLAG samples oxygenated by different methods is compared amongst each other, including reproduced results on overdoped PLD films by A. Stangl et al.^18^. As it is observed from the first glance itself, $T_c$, *c*-axis, and $n_H$ correlate well with each other. In Figure 4a , in the underdoped regime, $T_c$ is rather low and then sharply increases to 90 K as the charge carrier density increases towards the optimally doped value $n_H = 3.2\times 10^{21} cm^{-3}$. At this optimally doped point, $T_c$ is maximum, reaching 92 K. Figure 4b illustrates a complementary tendency in the relationship between the charge carrier density and the *c*-axis. In the underdoped state, the *c*-axis is highly impacted by $n_H$ and the crystal structure of YBCO undergoes a considerable change when it transitions from underdoped to optimally doped, which will have an impact on the superconducting properties. However, beyond the optimal doping, when $n_H$ keeps increasing, the *c*-axis and $T_c$ remain almost constant through the overdoped regime. This tendency is true for YBCO films grown via different processes (PLD, and TLAG), indicating that it’s a general pattern that holds regardless of the material’s growth process^48^.

**Fig. 5:**
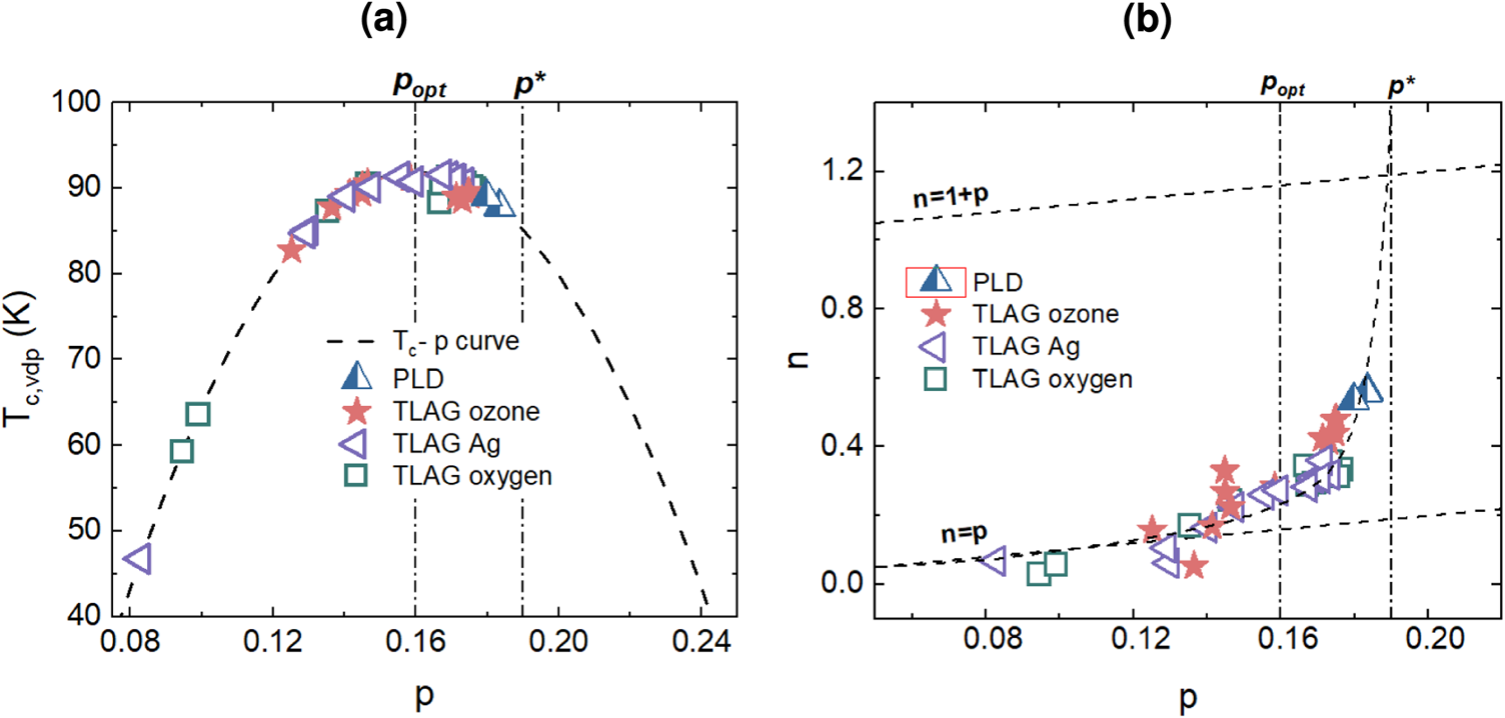
Doping state comparison between TLAG and PLD YBCO films: (**a**) Phase diagram of YBCO relating $T_c$ and $\textit{p}$ by the parabolic relation ${1-T_c/T_{c,max}=82.6(p-0.16)^2}$ with $T_{c,max}$ = 92 K. Samples grown via PLD and TLAG are placed on the phase diagram. For underdoped samples, $\textit{p}$ is calculated from $T_c$ using the parabolic relation. For optimally doped and overdoped films, $\textit{p}$ is calculated from the *c*-axis (described in methods). (**b**) Relationship between $\textit{p}$ and charge carrier density per Cu atom, n at 100 K ($n=n_HV/2, V=0.173nm^3$). Below $\textit{p}_{opt}$, the samples tend to follow $n=p$ behavior, but beyond this point a transition occurs to $n=1+p$. The dashed lines following the data points is a guide to the eye. The optimal doping $\textit{p}_{opt} = 0.16$ and the critical doping $\textit{p}^* = 0.19$ are represented by vertical lines in both the figures.

Figure 5a depicts the relationship between $$T_c$$ and the calculated $$\textit{p}$$. It is clearly observed from this figure that the TLAG films fall under a broad region starting from underdoped to beyond the optimal doping $$\textit{p}=0.16$$ till finally approaching the critical doping $$\textit{p}^*=0.19$$ where the maximum critical current density is expected. Along with the TLAG films, the overdoped PLD films reproduced from^18^ are also shown for comparison purposes. The critical temperature $$T_c$$ follows the well-known parabolic relationship with $$\textit{p}$$. Even though there is more distinction of doping state with $$\textit{p}$$ than $$T_c$$ and *c*-axis, the points are clustered between the optimal doping and critical doping, and TLAG films fall below $$\textit{p}^* = 0.19$$. In Figure 5b , we have calculated the charge carrier density per Cu atom, $$n={n_HV/2}$$ and plotted it against the hole concentration $$\textit{p}$$. Initially, an increase in $$\textit{p}$$ causes a proportional increase in charge carrier density per Cu atom. Interestingly, beyond the optimal doping $$\textit{p}_{opt} = 0.16$$, a steep increase in *n* values up to the critical doping $$\textit{p}^* = 0.19$$ is observed. This kind of behavior has been attributed to the Fermi surface reconstruction happening close to the critical doping $$\textit{p}^*$$^22^. This sharp rise demonstrates how intricate and nonlinear the doping process is, with minor adjustments to the doping concentration leading to notable changes in the electronic and superconducting characteristics of the material.

**Fig. 6:**
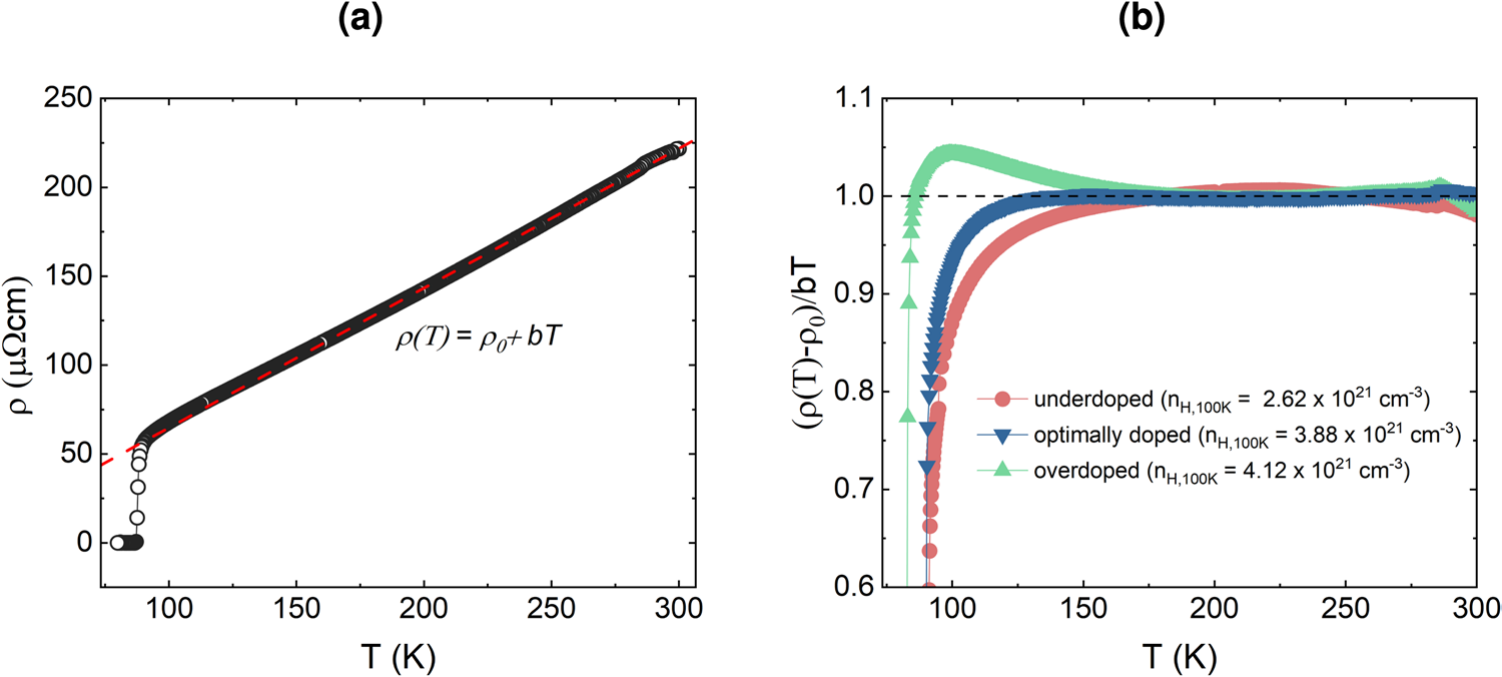
Normalized resistivity analysis: (**a**) Exemplary $\rho (T)$ plot for a sample with linear fitting ${\rho (T)=\rho _0+bT}$ for $\rho (T)>150\,K)$ (**b**) Normalized resistivity ${(\rho (T)-\rho _0)/bT}$ for an underdoped, optimally doped, and overdoped TLAG YBCO film; their corresponding $n_H$ values at 100 K are also given in the legend. The horizontal line at $y=1$ represents the normal state linear behaviour of resistivity.

A further indication of overdoping in our samples was observed from the behavior of the normal state resistivity in these films. In cuprates, the normal state resistivity follows a linear relationship with temperature that can be expressed as follows.

1 $$\begin{aligned} {\rho (T)=\rho _0+bT} \end{aligned}$$

where *b* is the slope which depends on the doping state^27,48^ and $\rho _0$ is the intercept, which is the resistivity at 0 K. An example fit using Equation 1 for the temperature range between 150 K and 300 K is shown in Figure 6a . Below 150 K, as the superconducting transition is approached, $\rho$ begins to deviate from the linear behavior depending on the doping state. This deviation is more clearly seen in the normalized resistivity ${(\rho (T)-\rho _0)/bT}$ plot shown in Figure 6b .

When the resistivity follows a linear relationship with temperature, the value of ${(\rho (T)-\rho _0)/bT}$ equals 1. However, near the transition temperature, some charge carriers migrate into the pseudogap and no longer participate in normal electrical transport^49^. This phenomenon causes differences between doping states: in an underdoped film, the normalized resistivity deviates downward due to a lack of charge carriers, while in an overdoped film, the deviation is upward. Figure 6b shows three films with underdoped, optimally doped, and overdoped behavior, consistent with their $n_H$ values indicated in the label. This analysis, along with other parameters discussed earlier, confirms overdoping in TLAG films. Since the direct evaluation of the oxygen content in YBCO is a cumbersome process, the above doping state analysis provided us with an indirect estimation of the doping state. The overall analysis indicates that the TLAG films are overdoped through the various oxygenation methods used.

### Impact of doping state on critical current density $J_c$

**Fig. 7:**
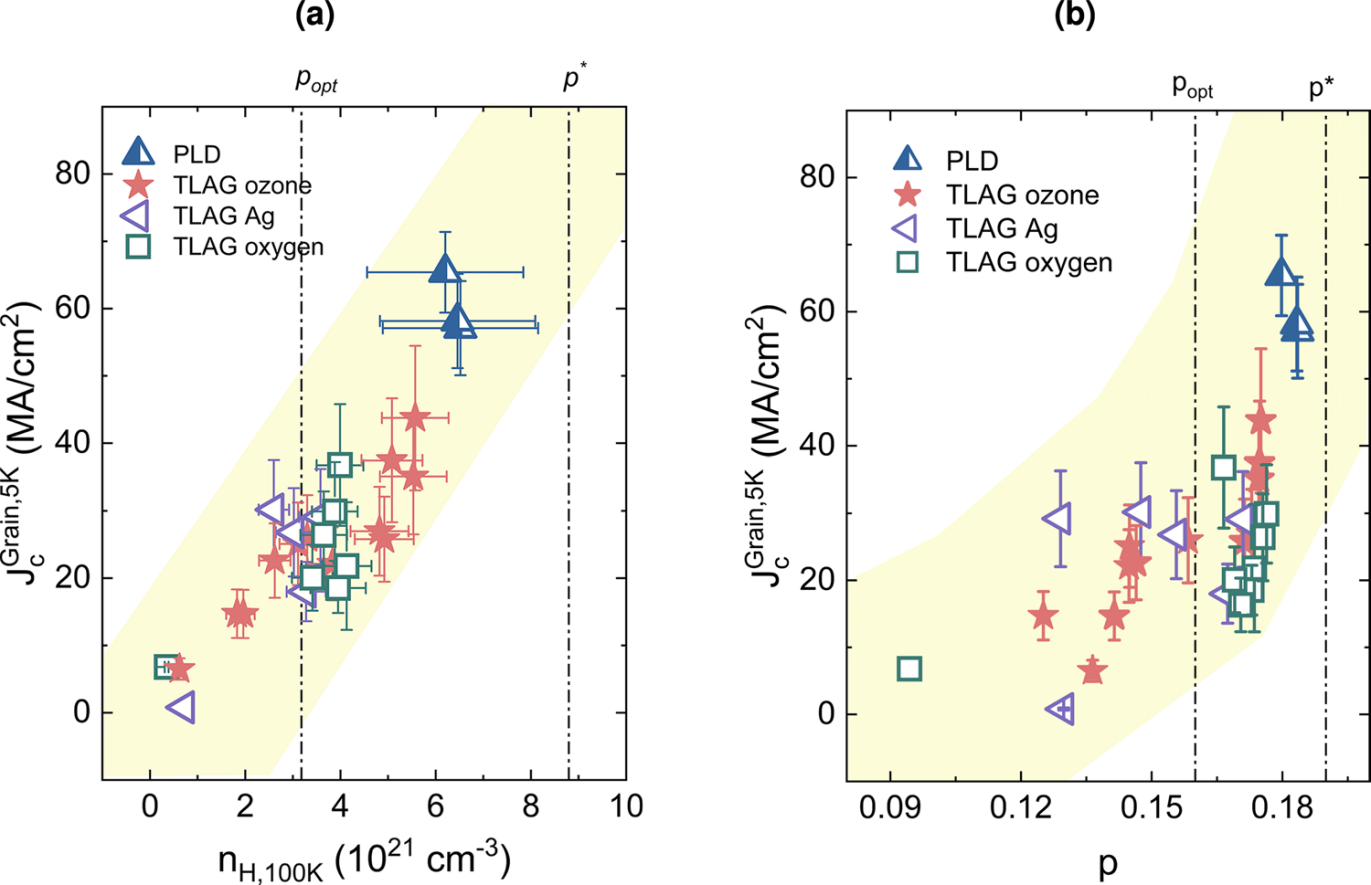
Analysis of critical current density in TLAG films as a function of doping parameters: (**a**) Hall carrier density $n_H$ and (**b**) doping level $\textit{p}$. The results obtained by A. Stangl et al.^18^ on PLD films are reproduced and also shown for comparison purposes.

The ultimate aim of all the investigations shown above is to understand the impact of overdoping on the critical current density. In this section, the effect of overdoping on the critical current density in TLAG films will be analyzed and compared to that of PLD-grown films. Our comprehension of the possibility of overdoping in TLAG films will be strengthened by this analysis, especially in light of prospective future applications. The evolution of the grain critical current density as a function of $n_H$ and $\textit{p}$ is depicted in Figures 7a and 7b . The anticipated increase of critical current density with overdoping is observed. Amongst all three oxygenation methods explored, ozone enables the achievement of higher $n_H$ values. The maximum $n_H$ value that we obtained with ozone-assisted oxygenation is in the range of $6\times 10^{21} cm^{-3}$ at 100 K. However, this enhancement is not always reflected in the $J_c$ values, probably due to the deterioration of the surface (see supplementary information, Figure S4) and the chlorine intergrowth observed, which probably arises from using the inappropriate gas tubing, and thus could plausibly be eliminated.

We observe that the PLD-grown films, oxygenated as explained in the methods, are able to achieve higher values of $J_c$ and $n_H$. The evolution of $J_c^{\text {Grain}}$ with $\textit{p}$ shows distinguished behavior as observed in Figure 7b . Initially, a slow linear increase up to the optimal doping $\textit{p}_{opt}$ and then a fast increase with highly dispersed values of $J_c^{\text {Grain}}$ increase towards the critical doping $\textit{p}^*$ is observed. This again corroborates the complexity of increasing the critical current density by tuning the overdoped state. Although the overdoping of TLAG films is confirmed by the $n_H$ and *c*-axis values, an increase in critical current density is a more complicated issue; the ultra-high values obtained in PLD films by A. Stangl et al.^18^ could not be achieved in TLAG films up to now. We attribute this primarily to the microstructural differences between PLD and TLAG-grown YBCO films. PLD is a well-established technique that can produce highly clean and homogeneous microstructures with minimal secondary phases and high epitaxial quality (see for example supplementary information Figure S6 for microstructure of our PLD films). This is reflected in the Δω values from rocking curves; PLD films exhibit Δω in the range of 0.1º - 0.12º^50^, whereas in TLAG films, Δω is slightly higher in the range of 0.2º - 0.3º^8^. The microstructure of our PLD-grown YBCO films mainly contains coherent twin boundaries and dispersed 2-5 nm Y_2_O_3_ or Y_2_Cu_2_O_5_ nanoinclusions that can possibly act as effective artificial pinning centres. In contrast, TLAG generates a more complex microstructure due to its ultrafast growth kinetics. This includes stacking faults, twin boundaries, and local strain inhomogeneities, and, being a relatively new process, their distribution may not be fully controlled. Additionally, in the case of TLAG ozone films, the presence of the thick planar defects impedes the current flow. Direct, quantitative comparisons of defect landscape and oxygen incorporation efficiency between these methods remain at an early stage and require further systematic study, representing a key direction for ongoing research. Overall, even though the fine-tuning and precise quality control of TLAG-grown films are still under investigation, we demonstrated here the capability of TLAG YBCO films in achieving the overdoped state.

### Overdoping in TLAG nanocomposites

One strategy to overcome the limitation of achieving high critical current density in TLAG YBCO films is to extend overdoping into nanoengineered films with APCs, where the increase of pinning force by overdoping is expected to have more impact^14^. We briefly explored this possibility in this work, and the preliminary results are shown in this section. TLAG nanocomposite (NC) YBCO films were grown and oxygenated with pure oxygen (TLAG NC oxygen) and with the aid of Ag islands on top (TLAG NC Ag) under the same conditions where we obtained overdoping in pristine films.

**Fig. 8:**
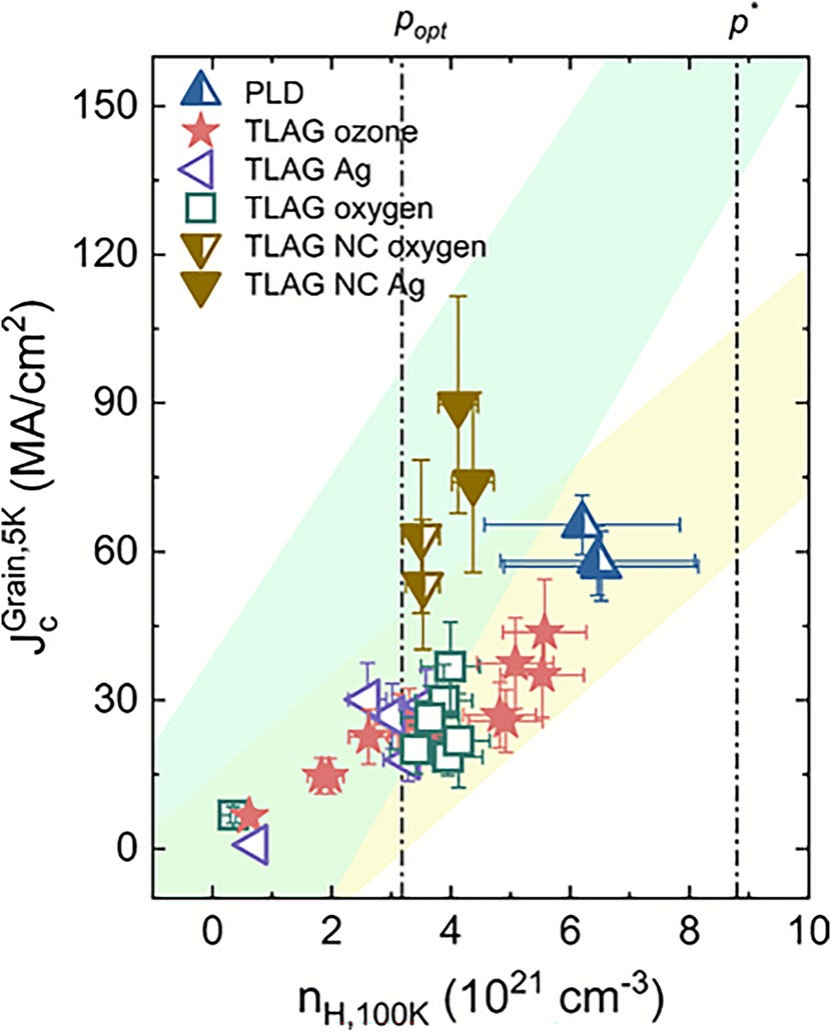
Overdoping of TLAG nanocomposites: The results of overdoping in TLAG nanocomposites, along with TLAG pristine, and PLD samples from Figure 7. TLAG nanocomposites show higher critical current density for the same range of $n_H$ values as TLAG pristine.

The results are shown in Figure 8. For similar values of $n_H$, TLAG nanocomposites achieve higher $J_c^{\text {Grain}}$ values than any pristine films, especially with the help of Ag islands. We envisage that this enhancement is due to the presence of a high density of pinning sites in nanocomposites, for instance, nanoparticles, twin boundaries, and nanostrain associated with stacking faults^51^. The corresponding increase in the condensation energy by overdoping impacts the elemental pinning force of each of these pinning centers^14^. Our findings closely agree with the work by M. Miura et al.^21^, where they demonstrated an increase of the critical current density $J_c$ in nanoengineered REBCO films in the overdoped regime.

These preliminary findings on overdoping of TLAG should be further investigated to reach highly overdoped TLAG YBCO nanocomposite films with high percolative critical current density. Additionally, it has to be remembered that the oxygenation process has significant dependence on the microstructure of the films, and since TLAG nanocomposites possess a strongly different microstructure (shown in supplementary information Figure S7), the optimal oxygenation conditions could vary. In the scope of this work, we highlight the potential application of overdoping in nanoengineered films as a robust and scalable manner to increase performance, which motivates further investigation into overdoping of REBCO nanocomposite films and CC.

## Conclusion

In this study, we used the novel growth technique TLAG combined with the chemical solution deposition approach to grow YBCO films. We then oxygenated the films by different methods in order to overdope and analyze their doping state properties. By using pure oxygen, ozone-oxygen mixture, and a Ag decoration layer for oxygenation, we were able to successfully create overdoped TLAG YBCO films. We also verified that the dependence of critical current density on charge carrier density holds true in the case of TLAG YBCO films. However, TLAG films did not surpass the $J_{c}$ of PLD-grown films, possibly due to differences in the underlying microstructure between the two types of films, which should be investigated in future studies..

In conclusion, the study highlights the unique advantages of TLAG films, particularly their ability to achieve overdoping, which is crucial for optimizing the superconducting performance of YBCO films for advanced applications. In addition to improving our understanding of the TLAG process, knowing the precise doping state and critical current density dependence of these films offers important insights for customizing materials to satisfy technical requirements in the next technological developments.

By combining TLAG’s fast growth kinetics with nanoscale defects and systematic overdoping, we achieved a bigger boost in $J_c$ than from any one method alone. This shows how defect density plays a crucial role in determining the efficacy of overdoping and highlights TLAG nanocomposites as a strong candidate for overdoping to improve the superconducting properties.

## Methods

YBCO pristine and nanocomposite (YBCO-BaZrO$_3$) films were grown by fluorine-free Chemical Solution Deposition (CSD) combined with Transient Liquid Assisted Growth (TLAG)^6–10^. A metal organic propionate-based solution containing Y, Ba, and Cu in excess is deposited on 5x5 mm SrTiO$_3$ (STO) substrates by spin coating, followed by pyrolysis treatment at $500\,^\circ \textrm{C}$ to obtain the nanocrystalline precursor layer composed of BaCO$_3$, CuO, and Y$_2$O$_3$. Uniform *c*-axis-oriented YBCO is grown from this amorphous film following a TLAG $P_{O_2}$-route process with growth temperatures between $800\,^\circ \textrm{C}$ and $900\,^\circ \textrm{C}$ and oxygen partial pressures of 1-2 mbar through the formation of a transient liquid.

These grown films then undergo the different oxygen annealing treatments for 60 min dwell time at the maximum annealing temperature. For TLAG oxygen films, post-annealing in a tubular furnace with a flow of oxygen of 0.6 L/min at 1 bar is performed. In the case of TLAG ozone, the oxygen line before entering the tubular furnace is split into two, and in one of the lines, an ozonizer is connected, which converts oxygen partially into ozone. The total flow in this line was reduced to 0.2 L/min due to the limitation on allowed pressure inside the ozonizer. Only with this ozone line, we could achieve ozone concentrations of 0.2 %, 0.4 %, 0.8 %, 1.2 %, and 1.9 %. With the help of the other line, which contains only oxygen, we diluted and achieved ozone concentrations below 0.2 %. YBCO films with an Ag layer (TLAG Ag) on top were prepared by sputtering 100 nm Ag squares of dimension 30x30 $\mu m$ all over the surface before oxygen annealing^27^. For reproducing the overdoped PLD films, we performed post-growth in-situ oxygenation inside the PLD chamber while cooling down from the deposition temperature to room temperature and around $600\,^\circ \textrm{C}$, a $P_{O_2}$ of 1 bar is applied.

The oxygenated films underwent structural, microstructural, magnetic, and electrical characterizations. Structural characterization was carried out using a Bruker D8 Discover diffractometer (Cu K$\alpha$, X-ray energy = 8.049 keV). The *c*-axis cell parameter is calculated by the Nelson-Riley method from the High-Resolution XRD pattern. For morphological characterization, scanning electron microscopy was carried out with a field emission QUANTA 200 FEG from FEI Company$^{TM}$, equipped with energy dispersive X-ray spectroscopy (EDX). For the microstructural study, cross-sectional TEM lamellae were prepared using a Thermo Fisher Scientific Helios 5 UX focused ion beam (FIB). The microstructure was analyzed using a double aberration-corrected Thermo Fisher Scientific Spectra 300 scanning transmission electron microscope (STEM), equipped with a Super-X EDX detector and operated at an accelerating voltage of 300 kV.

The doping state $\textit{p}$ was calculated from the *c*-axis parameter for both optimally and overdoped films using the empirical formula $\textit{p} = c_1y + c_2y^6 + p_0$ with $y = 1 - c/c_0$^52,53^. We used values from^53^ ($c_0 = 11.8447, c_1 = 11.491, c_2 = 5.17.10^9$) in this study. Adding the extra parameter $\textit{p}_0 = -0.027$ corrected a slight systematic constant offset.

Using the Bean critical state model for a thin disc, the critical current density $J_c$ was determined from the magnetization loop using Quantum Design’s SQUID magnetometer. For the electrical transport analysis, the Physical Property Measurement System (PPMS) by Quantum Design was used. Four contacts were attached to the corners of the films using Ag paint for measurements in the Van der Pauw configuration to calculate $n_H$ and $T_c$^54,55^. For $n_H$, the Hall voltage was measured as a function of magnetic field from -9 T to 9 T at 100 K, and for $T_c$, the temperature was swept from 300 K to a few Kelvin below the transition temperature.

## Supplementary Information

Supplementary Information.

## Data Availability

The data that support the findings of this study will be made available at the institutional repository “digital.csic” upon reasonable request.
