# Supplementary material for: Exploring the overdoping effects in Transient Liquid Assisted Grown YBa[image]Cu[image]O[image] superconducting films
Source: Sci Rep. 2026 Apr 1;16:15607. doi: 10.1038/s41598-026-41613-0 (PMC13187026; doi:10.1038/s41598-026-41613-0)
Supplement: Supplementary file 1 — Supplementary Information. [file 41598_2026_41613_MOESM1_ESM.pdf]

Exploring the Overdoping Effects in Transient Liquid Assisted Grown $YBa_2Cu_3O_{7-\delta}$ Superconducting Films

Aiswarya Kethamkuzhi[1,*], Lavinia Saltarelli[1], Kapil Gupta[2], Carla Torres[1], Diana Garcia[1], Joffre Gutierrez[1], Xavier Obradors[1], and Teresa Puig[1,*]

[1]Institute of Materials Science of Barcelona (ICMAB-CSIC), Spain
[2]Catalan Institute of Nanoscience and Nanotechnology (ICN2), Spain
[*]akethamkuzhi@icmab.es, teresa@icmab.es

Supplementary Information

(a)

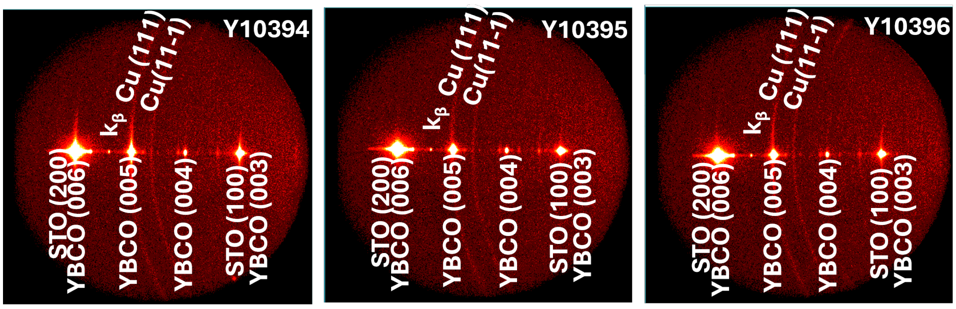

(b)

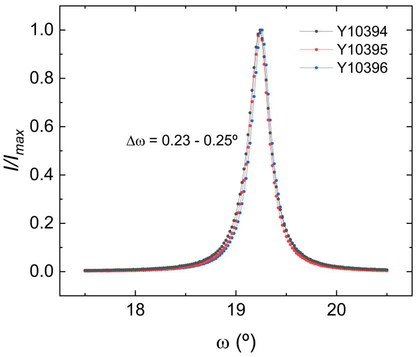

(c)

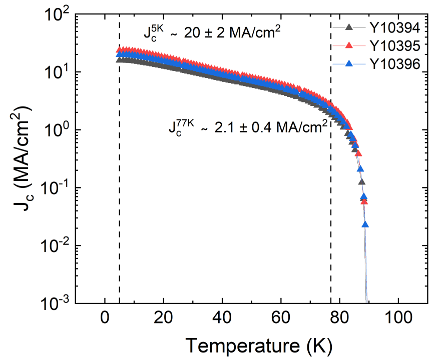

Figure S1: Extent of sample reproducibility: (a) The 2D-XRD images taken by GADDS (General Area Detector Diffraction System) from three TLAG YBCO films (Y10394, Y10395, and Y10396) grown and oxygenated under the same conditions. The confined spot of YBCO($00l$) reflections confirms the highly epitaxial c-axis orientation. (b) Rocking curve scan of YBCO (005) reflection using high-resolution XRD measurements to probe the out-of-plane orientation; the three samples shows $\Delta\omega = 0.23ž - 0.25ž$ which represents highly epitaxial orientation. (c) The resulting critical current density J_c as a function of temperature for the three samples. The values are reproducible within an error bar of 20%. This error bar is assigned to all the data points in our experiments.

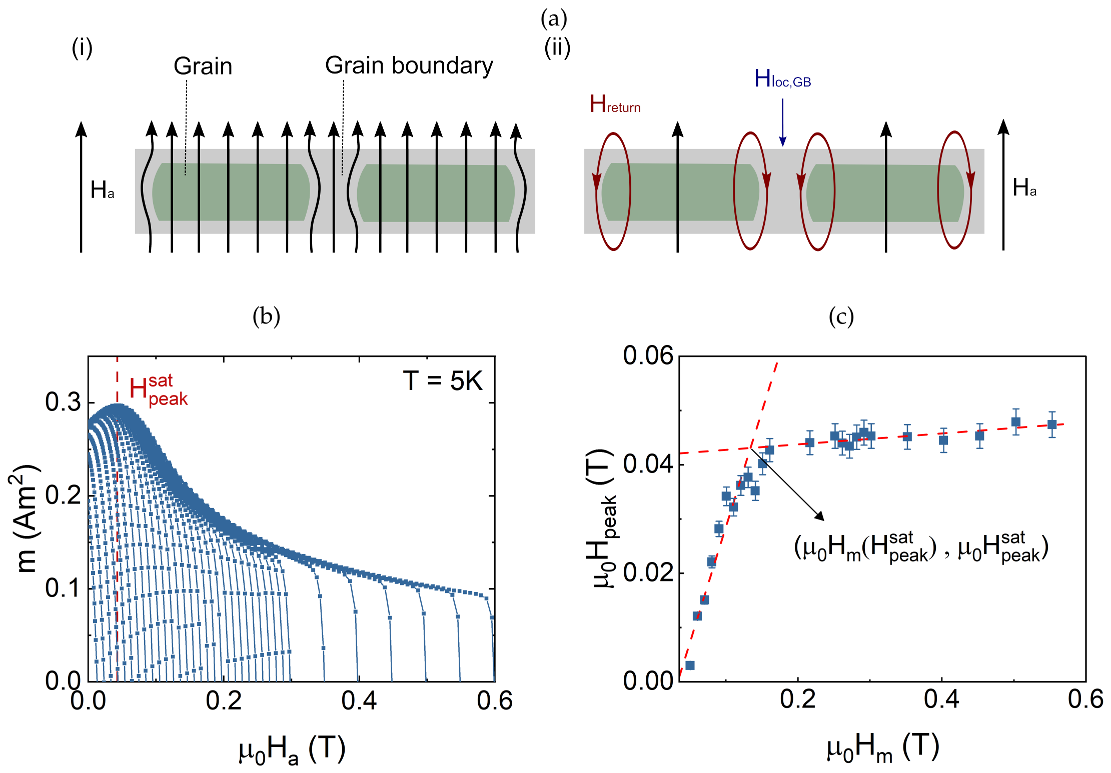

Figure S2: Determination of grain critical current density J_c^{Grain}: The grain critical current density (J_c^{Grain}) in YBCO films is evaluated using the inductive, non-invasive magnetometric technique from the work of A. Palau et al. [1] (a) In a granular sample, when a magnetic field H_a is applied, the field lines penetrate both the grains and the grain boundaries, as shown in (i). As the magnetic field is decreased during the reverse branch of the hysteresis loop, the trapped field in the grains returns through the grain boundaries, whose superconducting properties are not as strong as those of the grains, creating a return magnetic flux H_{return} (ii). This process generates a local magnetic field at the grain boundaries opposite to the direction of the applied magnetic field, which modifies the local field at the grain boundary, $H_{\text{loc, GB}} = H_a - H_{\text{return}}$. The magnetization reaches a maximum when $H_{\text{loc, GB}} = 0$, i.e., when $H_a = H_{\text{return}}$, giving rise to the characteristic peak in the reverse branch as shown in (b). Since the loops start from low H_a, insufficient to fully magnetize all grains, the peak position H_{peak} shifts with increasing maximum applied field (H_m) until saturating at $H_{\text{peak}}^{\text{Sat}}$. (c) The plot of H_{peak} against the maximum applied field H_m for each loop; the model relates the saturation magnetic field $H_{\text{peak}}^{\text{Sat}}$ and the corresponding maximum magnetic field applied $H_m(H_{\text{peak}}^{\text{Sat}})$ to the J_c^{Grain} as: $J_c^{\text{Grain}} = \frac{H_m(H_{\text{peak}}^{\text{Sat}})}{2f_1 D}$, $\frac{H_{\text{peak}}^{\text{Sat}}}{H_m(H_{\text{peak}}^{\text{Sat}})} = f_2$, where D is the average grain diameter and f_1, f_2 are numerically derived dimensionless factors that depend on D/t (t is the film thickness).

(a)

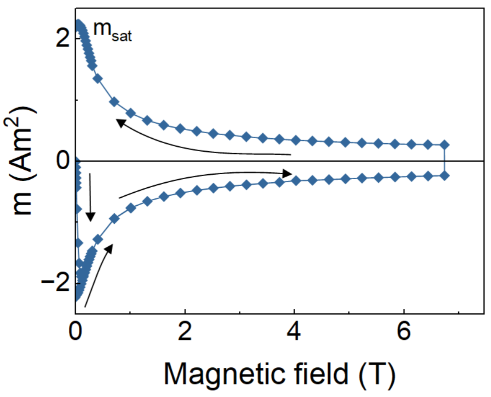

(b)

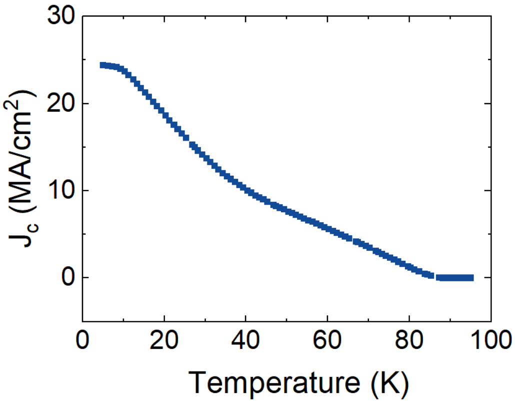

Figure S3: Determination of J_c from SQUID magnetization measurements: The Bean critical state model provides a direct relationship between the critical current density J_c and the saturated magnetization m_{sat} of a superconducting sample. (a) The m-H hysteresis loop at 5 K is obtained by increasing the magnetic field from zero to 7 T and then decreasing the field back to zero. This process results in a trapped magnetic flux state, or Bean critical state, with remanent magnetization m_{sat}. The hysteresis loop is continued by applying the negative field of -7 T and then increasing the field back to zero. The separation between the branches corresponding to increasing and decreasing magnetic fields is proportional to the critical current density J_c according to the Bean critical state model. (b) The temperature dependence J_c by applying the Bean critical state model in the remanent state for a thin disc [2]; $J_c = \frac{3\Delta m}{2\pi r^3 t}$, where Δm is the full width of the saturated hysteresis loop, t is the film thickness, and $r = \sqrt{a^2/\pi}$ is the effective radius of the square samples with side length a.

References

[1] A. Palau et al. "Simultaneous determination of grain and grain-boundary critical currents in superconductors". In: *Physical Review B* 75.5 (2007), p. 054517. DOI: 10.1103/physrevb.75.054517.

[2] D-X Chen and Ronald B Goldfarb. "Kim model for magnetization of type-II superconductors". In: *Journal of Applied Physics* 66.6 (1989), pp. 2489–2500.

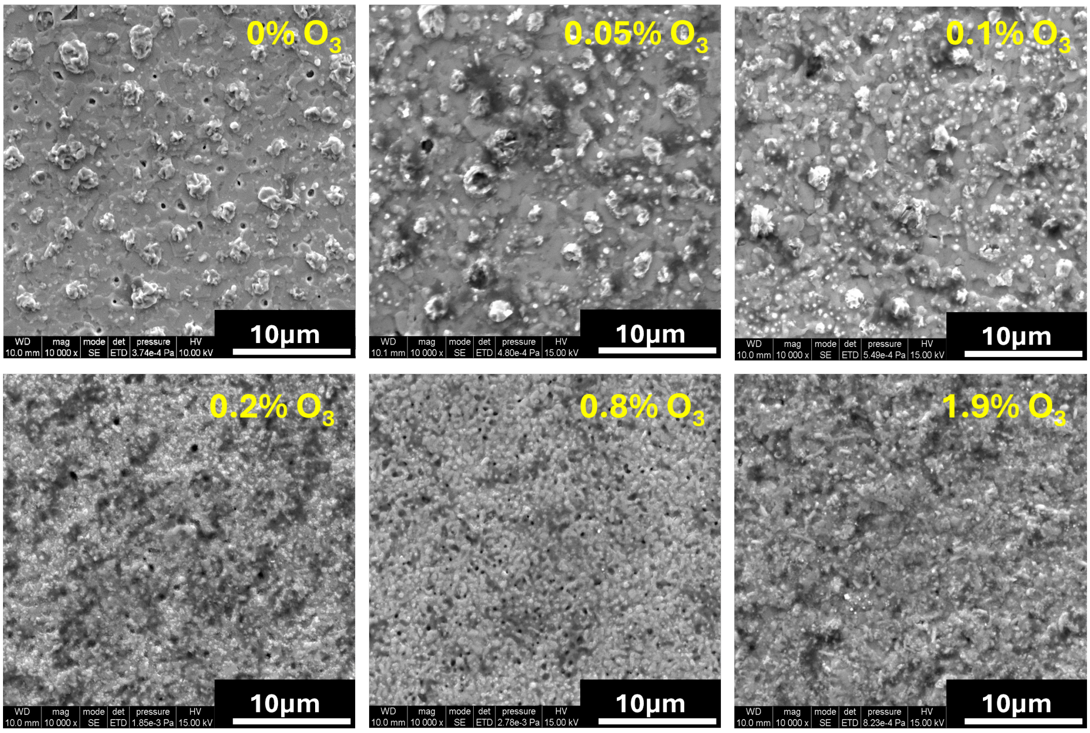

Figure S4: Surface degradation due to ozone: Evolution of the surface morphology of YBCO films treated with different concentrations of ozone through SEM images; gradual degradation of the surface is visible. Notice that the flat surface with the CuO precipitates (the latter due to the Cu-excess solution used for TLAG films) disappears at high ozone concentrations due to the surface degradation.

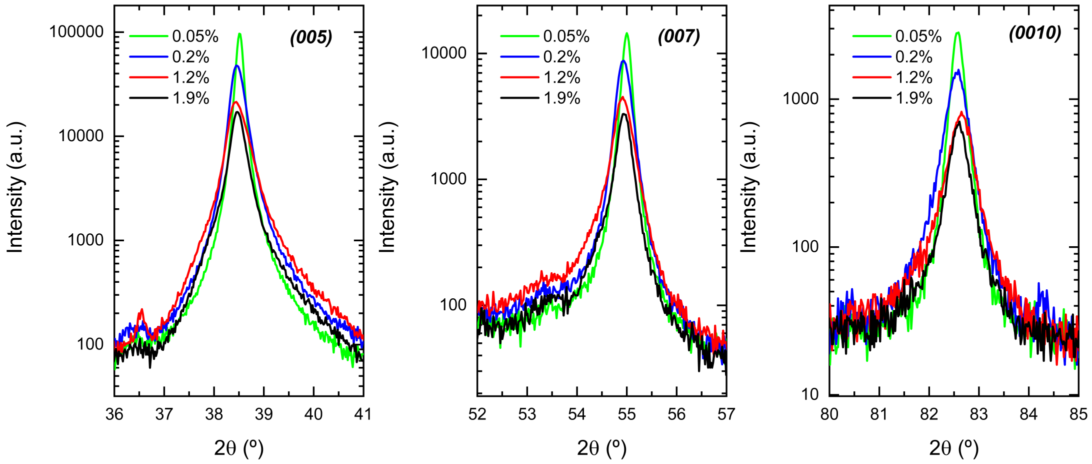

Figure S5: X-ray diffraction pattern of *(00l)* peaks of YBCO showing higher concentration of ozone causes a decrease in the intensity of the peaks.

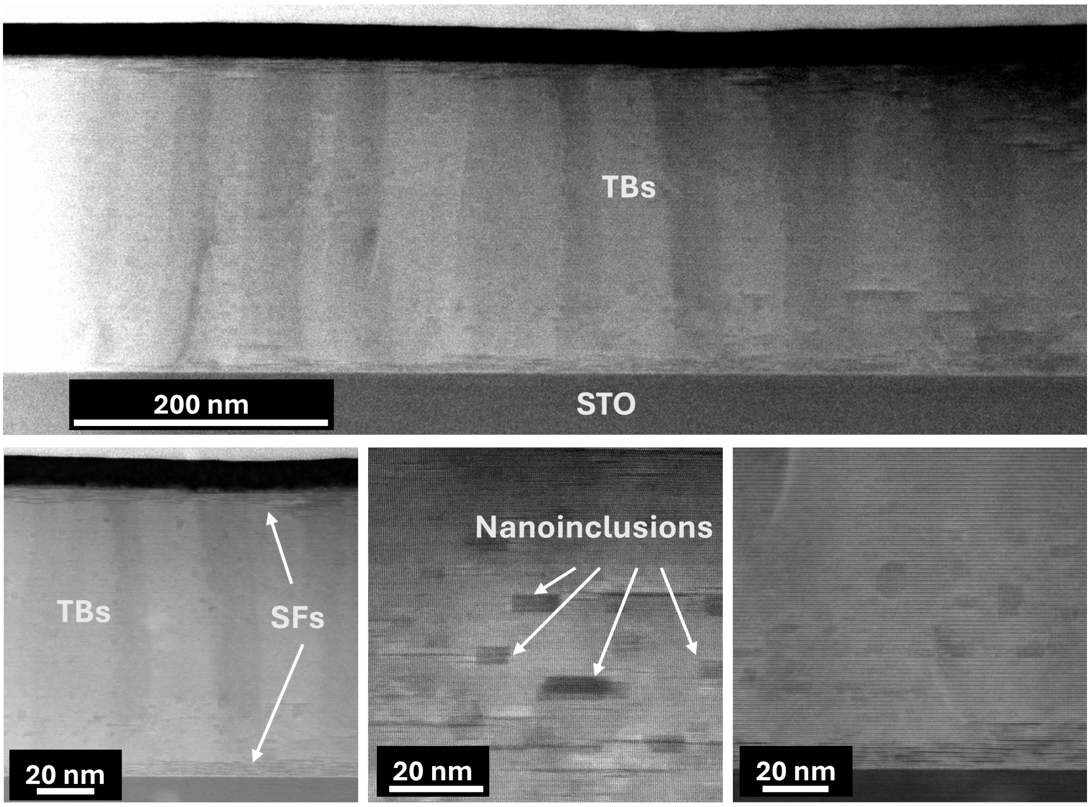

Figure S6: Microstructure of the PLD-YBCO film of this work, largely free of extended defects, exhibiting only a limited number of short stacking faults localized near the surface and interface. The PLD-grown films also displays coherent twin boundaries and contain 2-5 nm sized Y_2O_3 or $Y_2Cu_2O_5$ nanoinclusions, both of which can act as effective pinning centers.

(a)

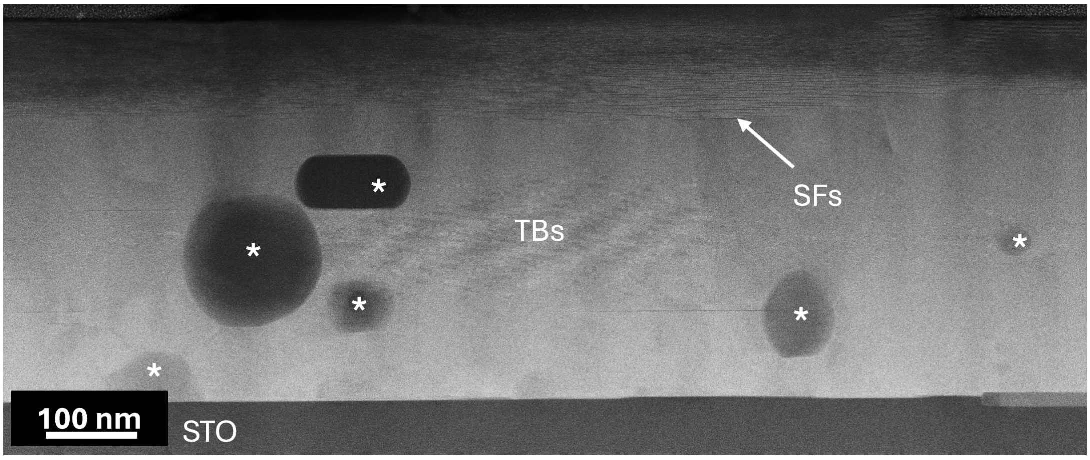

(b)

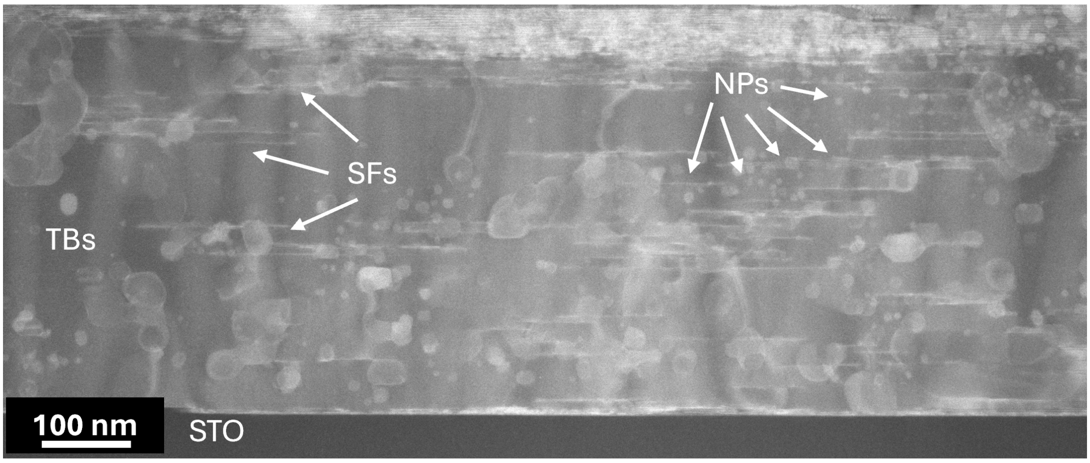

Figure S7: Microstructure comparison between TLAG pristine and nanocomposite film: (a) The STEM-HAADF (Scanning Transmission Electron Microscopy - High Angle Annular Dark Field) image of a TLAG pristine film oxygenated with Ag surface decorative layer; mainly contains coherent twin boundaries (TBs) and few stacking faults (SFs) on the top. (b) The STEM-HAADF image of a TLAG nanocomposite film (YBCO with $BaZrO_3$ nanoparticles) oxygenated with Ag surface decorative layer; the microstructure contains various types of pinning centers, including stacking faults (SFs), twin boundaries (TBs), and nanoparticles (NPs) of different sizes. Secondary phases are marked with asterisks. The most commonly occurring secondary phases are CuO, some Ba-Cu-O phase (eg: $BaCu_3O_4$), Y211 phase (Y_2BaCuO_5), Y_2O_3, etc.
